# Gender characteristics, social determinants, and seasonal patterns of malaria incidence, relapse, and mortality in Sistan and Baluchistan province and other province of Iran: A systematic review and meta-analysis

**DOI:** 10.1186/s12879-025-10542-0

**Published:** 2025-02-01

**Authors:** Maryam Khazaee-Pool, Mahmood Moosazadeh, Mehran Asadi-Aliabadi, Fereshteh Yazdani, Koen Ponnet

**Affiliations:** 1 Department of Health Education and Promotion, School of Health, Health Sciences Research Center, Mazandaran University of Medical Sciences, Sari, Iran; 2 Associate Professor of Epidemiology, Gastrointestitional Cancer Research Center, Non-Communicable Disease Institute, Mazandaran University of Medical Sciences, Sari, Iran; 3 Health Sciences Research Center, Mazandaran University of Medical Sciences, Sari, Iran; 4 Department of Communication Sciences, Imec-Mict-Ghent University, Ghent, Belgium

**Keywords:** Malaria, Gender, Social Determinants, Seasonal Patterns, Climate, Sistan & Baluchistan, Iran

## Abstract

**Introduction:**

Malaria is a climate-dependent disease influenced by gender, social determination, seasonal patterns, and relapse incidence This study reviews these characteristics of malaria in the Sistan and Baluchistan (S&B) province and another province of Iran.

**Methods:**

This systematic review and meta-analysis was conducted through systematic and manual searches in electronic databases such as PubMed, Web of Science, Scopus, Science Direct, Google Scholar, and the Scientific Information Database. Studies from the S&B province, published from 1990 to 2024, written in Farsi and English, and reporting incidence, relapse, or death were included in this study. The quality of the studies was evaluated using the Newcastle–Ottawa Scale.

**Results:**

Out of 1941 studies initially identified, 43 were included in the systematic review, with 12 studies on relapse and 43 on incidence included in the meta-analysis. The combined results of the 43 primary studies using the random effect model showed that the frequency of malaria among infected women is 32% in S&B, 22% in other parts of Iran and 24% in the whole of Iran. This is 68%, 77% and 75% in men respectively. Among the primary studies, 8 deaths were reported in two studies, all of which were men. The incidence rate of malaria relapse varied from 0.30% to 46%. Based on the random effect model, the malaria relapse rate in Iran was estimated at 9%. The highest incidence of malaria in Iran was between spring and summer, and the lowest incidence was winter and spring. Most studies showed a downward trend in malaria incidence of malaria between 1986 to 2019. In term of socio-demographic status, malaria was more common in rural areas (82%). Although 28% were employed, and in term of age, young and old individuals (15 to 50 years) had the highest rate of infection.

**Conclusion:**

This systematic review and meta-analysis revealed a lower incidence of malaria in women compared to men. The findings highlight the need for health care and the importance of targeted malaria control interventions, especially for men, rural areas, hot weather conditions, and young to middle-aged age groups, particularly in the S&B province.

**Supplementary Information:**

The online version contains supplementary material available at 10.1186/s12879-025-10542-0.

## Introduction

Malaria is a life-threatening disease caused by parasites that are transmitted to humans through the bite of infected female anopheles’ mosquitoes. Despite being preventable and treatable, in 2022, nearly half of the world’s population was at risk of malaria. Based on WHO there were an estimated 263 million cases of malaria in 2023, and the estimated number of malaria deaths stood at 597 000. While sub-Saharan Africa bears a disproportionately high share of the global malaria burden, the WHO regions of South-East Asia, Eastern Mediterranean, Western Pacific, and the Americas also report significant cases and deaths [1]. In Iran, malaria is endemic in Sistan and Baluchistan (S&B), Hormozgan, and Kerman provinces [2]. 77.4% and 78% of malaria cases from 2016 to 2022 were in the southernmost region of Iran [3]. Despite a decrease in annual reported malaria cases in Iran, the infection remains a critical health issue in areas such as S&B [4]. This region, as the largest province of southeastern Iran, lags in social, economic and cultural development compared to other regions [5]. In addition, its proximity to Afghanistan and Pakistan -both with high malaria incidence- contributes to the high malaria rates in S&B [6]. Although, S&B saw a more than 5-fold increase in malaria prevalence between 2022 and 2023 [3]. Therefore, malaria is still prevalent in this area and needs more attention to eradicate it. This is while malaria has reached the eradication stage in some province of Iran [7]. Hence, paying attention to the characteristics influence malaria incidence and comparing S&B province with other provinces of Iran can be beneficial.

Malaria transmission depends on a combination of factors [8]. In other words, several determinants influence malaria incidence, relapse, and mortality, including socio-economic status, cultural practices, and environmental factors. Among these, climate change is notably influential. The public health impact of climate change and the growing threat of antibiotic resistance are among the top global health challenges of the 21st century. Climate change is expected to have a variety of effects on human health. One of which is the transmission of infectious diseases through vectors—a factor that has been under-considered and is less predictable [9]. Infectious agents such as protozoa, bacteria, viruses, and related vector organisms (like mosquitoes) lack temperature regulation mechanisms making their reproduction and survival sensitive to temperature fluctuations[10]; This dependence on temperature is observed in the relationship between disease severity and weather changes over weeks, months, and years [11]. A warm and unstable climate plays a dynamic role in the global emergence, revitalization, and redistribution of infectious diseases [12]. Furthermore, the indirect impacts of climatic change such as increased natural hazards like floods and extreme weather conditions; also elevate the risk of infectious diseases [13].

There may be differences in demographic characteristics (gender, age), geographical location (altitude, temperature, rainfall), and differences in economic activities that have also affected the prevalence of malaria [14]. Malaria is one of the diseases that is claimed to be under both the direct and indirect impacts of climate change [15]. Climatic and environmental conditions play an important role in the life cycle, duration of activity, and proliferation of anopheles mosquitos [16], making malaria a climate-dependent disease. Due to climate change, malaria incidence is increasing in different regions of Iran [17]. Understanding the relationship between climate and malaria is essential, considering the complex and multidimensional impacts, including changes in the timing and location of transmitted diseases [18]. In endemic regions where Malaria transmission occurs seasonally or sporadically, it can lead to high mortality across all age groups [19].

Significant progress has been made in combatting infectious diseases of poverty, including malaria, particularly through large-scale, coordinated disease control programs. However, substantial public health challenges remain, ranging from global environmental issues to gender and intersecting inequalities affecting health conditions related to infectious diseases in low- and middle-income countries (LMICs). Malaria is still considered an important health problem in Iran [20]. Various regional and global evaluations of the effective models of malaria and climatic change have yielded different results [19]. A 30-year study (1975–2005) evaluating the climatic conditions for malaria outbreaks in Iran identified climatic factors as a risk factor for increased malaria outbreaks [21].

Although factors like urbanization, globalization, population movement, deforestation, and interruptions to control measures -as well as biotic factors such as human host characteristics- affect malaria incidence and severity [22], climate change represents a potential environmental factor influencing disease outbreaks [23]. In addition, gender role and position must be considered. Gender is a social determinant of health that influences a person’s risks from exposure and vulnerability to disease. Sex and gender are key drivers of health outcomes, affecting access to and delivery of health products and services for the prevention and control of infectious disease like malaria. All genders differ in their needs, perceptions, attitudes, and vulnerability to the effects of climate change. Despite clear disparities, gender-disaggregated health data are often either under-represented or non-existent when assessing the health effects of climate change in medical research, environmental studies, and strategic planning for mitigation and adaptation policies [24].

This disregard for gender differences is particularly concerning as climate change is predicted to worsen existing social and economic inequalities both between and within countries. This disparity is notably true for how climate change affects health [25]. Therefore, assessment of the trend of malaria prevalence is important in the control and prevention of the disease [14]. In Other words, understanding the intersection of different dimensions of gender with other social stratifies, including age, sex, among others, is critical in the epidemiology, prevention, and control of infectious diseases like malaria across different contexts. There is increasing yet insufficient evidence on how and why gender intersects with other key social factors to shape infectious disease conditions like malaria, influence vulnerability to illness, and affect experiences in accessing health care. In addition, considering malaria is a climate-dependent disease, and given the role of gender as well as the effects of social determination, seasonal patterns and relapse incidence of malaria, this study reviews the gender characteristics, social determinants, seasonal patterns and relapse incidence of malaria disease in the S&B province and other province of Iran. The results of this study can be available to relevant experts and officials for planning malaria eradication, especially in S&B province, and adopting cost-effective approaches based on factors affecting incidence, recurrence, and mortality of Malaria.

## Methods

### Design

This study is designed and conducted based on Preferred Reporting Items for Systematic Reviews and Meta-Analyses (PRISMA) guidelines [26]. Before data extraction, the study was registered in the International Prospective Register of Systematic Reviews (PROSPERO; Registration number: CRD42024568936)

### Literature search and search strategy

In the present study, the published studies were systematically searched by two authors between October 1990 and August 2024 across databases including ScienceDirect, Scopus, PubMed, Web of Science, Google Scholar, and the Iranian database, including the Scientific Information Database. The search was typically conducting using Persian and English keywords. In the PubMed databases, search terms were extracted using Medical Subject headings included Malaria, Climate change, Iran, and Gender. For manual searching and screening, we used keywords such as Income, Racial Groups, Social Status, Occupant, Education, Temperature, Humidity, Atmospheric pressure, Wind, Impact, Variability, Adaptation, and Mitigation. The systematic review search strategy is detailed in Table 1. all studies were reviewed for related publications. We also searched existing gray literature to strengthen the data, including reports from the Ministry of Health of Iran (annual reports, Research Reports, Technical Reports, Project Report), official government documents/reports, unpublished clinical trials, conference abstracts, graduate theses and dissertations. Screening, data extraction, and quality assessment were performed by two authors (FY and MK) with disagreements resolved through discussion with a third author (MM). In addition, the references of the studies were searched to find related studies and increase search sensitivity. All collected references were entered into reference management software (EndNote).

**Table 1:** Search strategy in databases

	**#**	**Keyword**	**Mesh terms**	**Syntax**	**N**	**Date**
**PubMed**	#1	Malaria	Marsh Fever Fever, Marsh Remittent Fever Fever, Remittent Infections, Plasmodium Infection, Plasmodium Plasmodium Infection Plasmodium Infections Malaria	(((((((Marsh Fever[Title/Abstract]) OR (Fever, Marsh[Title/Abstract])) OR (Remittent Fever[Title/Abstract])) OR (Fever, Remittent[Title/Abstract])) OR (Infections, Plasmodium[Title/Abstract])) OR (Infection, Plasmodium[Title/Abstract])) OR (Plasmodium Infection[Title/Abstract])) OR (Plasmodium Infections[Title/Abstract])) OR (Malaria[Title/Abstract])	98,508	28/8/2024
	#2	Iran	Islamic Republic of Iran Iran	(Islamic Republic of Iran[Title/Abstract]) OR (Iran[Title/Abstract])	58,502	“
	#3	Gender	Gender Identity Sex	(Gender Identity[Title/Abstract]) OR (Sex[Title/Abstract])	711,091	“
	#4	Climate change	Change, Climate Changes, Climate Climate Changes Climate Change Temperature Humidity Atmospheric pressure Wind	(((((((Change, Climate[Title/Abstract]) OR (Changes, Climate[Title/Abstract])) OR (Climate Changes[Title/Abstract])) OR (Climate Change[Title/Abstract])) OR (Temperature[Title/Abstract])) OR (Humidity[Title/Abstract])) OR (Atmospheric pressure[Title/Abstract])) OR (Wind[Title/Abstract])	877,880	“
	#5	#		#1 AND #2	471	“
	#6	#		#1 AND #2 AND #3	4	"
	#7	#		#1 AND #2 AND #4	31	"
**WOS**	#1	Malaria	Same as PubMed	((((((((TS = (Marsh Fever)) OR TS = (Fever, Marsh)) OR TS = (Remittent Fever)) OR TS = (Fever, Remittent)) OR TS = (Infections, Plasmodium)) OR TS = (Infection, Plasmodium)) OR TS = (Plasmodium Infection)) OR TS = (Plasmodium Infections)) OR TS = (Malaria)	112,878	28/8/2024
	#2	Iran	“	(TS = (Islamic Republic of Iran)) OR TS = (Iran)	131,779	“
	#3	Gender	“	(TS = Gender Identity) OR (TS = Sex)	960,013	“
	#4	Climate change	“	(((((((TS = (Change, Climate)) OR TS = (Changes, Climate)) OR TS = (Climate Changes)) OR TS = (Climate Change)) OR TS = (Temperature)) OR TS = (Humidity)) OR TS = (Atmospheric pressure)) OR TS = ( Wind)	5,688,941	“
	#5	#		#1 AND #2	573	“
	#6	#		#1 AND #2 AND #3	7	“
	#7	#		#1 AND #2 AND #4	43	“
**Scopus**	#1	Malaria	Same as PubMed	(TITLE-ABS-KEY (marsh AND fever) OR TITLE-ABS-KEY (fever, AND marsh) OR TITLE-ABS-KEY (remittent AND fever) OR TITLE-ABS-KEY (fever, AND remittent) OR TITLE-ABS-KEY (infections, AND plasmodium) OR TITLE-ABS-KEY (infection, AND plasmodium) OR TITLE-ABS-KEY (plasmodium AND infection) OR TITLE-ABS-KEY (plasmodium AND infections) OR TITLE-ABS-KEY (malaria))	148,613	20/4/2024
	#2	Iran	“	(TITLE-ABS-KEY (Islamic AND republic AND of AND Iran) OR TITLE-ABS-KEY (Iran))	191,422	20/4/2024
	#3	#		#1 AND #2	767	20/4/2024

### Inclusion and exclusion criteria

The review question will be in line with the population, interventions, comparators, outcomes, timing and study design (PICOTS). P: All people in S&B province and other province. I: Not applicable in this study. C: Not applicable in this study. O: Consequences of infection and death T: from 1990 until 2024. **S:** All types of studies except Case reports, case series and RCTs.

### Outcome measure

In this study, the outcome was a systematic assessment of relapse standard error and prevalence of women with malaria among all infected people in S&B and other part of Iran.

### Data extraction

A data extraction sheet using Microsoft Excel software was developed to extract relevant information for further analysis. The extracted data included the first author, publication year, province, total number of malarias, total number of malarias based on gender, frequency of mortality and morbidity according to gender, frequency of relapse according to gender, demographic characteristics of patient (residence place, age and job statues), the number of malaria cases based on the seasonal pattern was also recorded. The authors independently extracted data into a sheet. any discrepancy in the data extraction process, was resolved through consensus after repeating the process.

### Quality assessment

Two authors independently assessed the quality of the included studies. Disputes between the two authors was resolved by consensus or the decision of a third author. The Newcastle–Ottawa Scale (NOS) for cross-sectional studies was used to evaluate the methodological quality of the included studies. The NOS for cross-sectional studies investigates three parameters (selection, comparability, and outcome), through eight particular questions. Each question in this scale is scored with one point, except for the comparability domain, in which each item is scored up to two points. Therefore, the quality of studies is classified into four groups: very good (9-10 points), good (7-8 points), satisfactory (6-5 points), and unsatisfactory (below 5 points).

### Data analysis

The standard error of the relapse and incidence in women for each of the primary studies is calculated based on the binomial distribution. Heterogeneity between the results of the primary studies is evaluated with the I-square and Q indices. Publication bias is assessed by the Egger’s test and funnel plots. Also, sensitivity analysis checks the impact of each of the primary studies on the overall estimate. Due to the existence of heterogeneity, random effect models and inverse variance methods were used to combine the results of the primary studies. The results of the primary studies will be presented in a forest plot diagram. It should be noted that a Trim and Fill test has been used to estimate the number of primary studies with publication bias. Data analysis is conducted using Stata Ver. 11 done.

### Ethics

The study procedure was approved by the Medical Ethics Committee of Mazandaran University of Medical Sciences [Grant NO: 18934; Ethical code number: IR.MAZUMS.REC.1402.451].

## Results

### Results of search and selection strategy

**Fig. 1:**
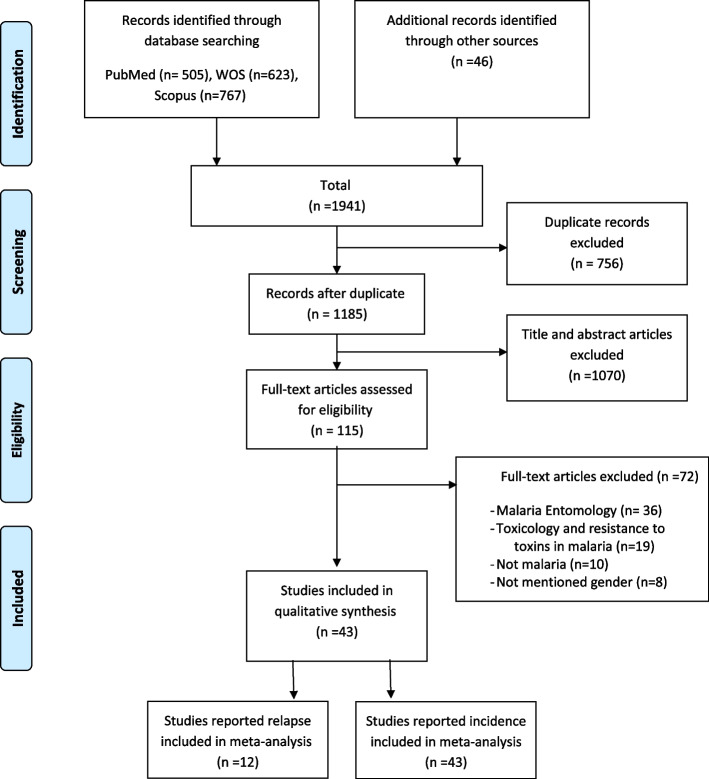
The PRISMA diagram for the search of records and study selection

The systematic search in the databases yielded a total of 1941 results (PubMed = 505, WOS = 623, Scopus = 767 and other sources = 46). After removing duplicate records using EndNote (*n* = 756), 1185 studies were retained. thereafter, 1070 studies were excluded because of a non-relevance title or abstract. Further, 72 studies were not retained because there was no association with malaria incidence, or gender, malaria entomology, and malaria-related toxins were not mentioned. Finally, 43 studies were used in this systematic review (Fig. 1).

### The characteristics of the included studies

Of the 43 included studies, 10 studies were conducted in the S&B region and 33 studies were conducted in Iran and other cities of Iran. The included studies were published between 1990 and 2024. The characteristics of the included studies are shown in Table 2. The quality of 43 studies was assessed using the NOS for cross-sectional studies. Among them, 18 studies had very good, 23 studies had good, and two studies had satisfactory methodological quality (Table 3).

**Table 2:** The number of malarias based on the gender, death and recurrent

Author, year	Place	Row	Men	Total Case of malaria	Year of review	Province, City	Recurrent		Death		Women
							**Female**	**Male**	**female**	**Male**	
Salehi, 2010 [7]	S & B	1	18,195	28,172	2005–2008	S & B	N^a^				9977
Salehi, 2008		2	27,700	42,162	2001–2006	S & B	N^a^				14,461
Sargolzaie, 2014		3	8880	13,620	2008–2011	S &B	N^a^				4740
Zayeri, 2011 [36]		4	42,695	64,920	2001–2009	S & B	N^a^				22,231
Youssefi, 2011		5	950	1464	2009–2010	Sarbaz	N^a^				514
Mehdipour, 2013		6	1572	2222	2007–2011	Saravan	N^a^				650
Mirahmadi, 2020 [4]		7	586	802	2001–2016	Khash	N^a^				216
Nili, 2023 [6]		8	3076	4074	2000–2019	Zahedan	N^a^				998
Sharifi-Mood, 2012		9	4183	7035	2006–2010	Nikshahr	N^a^				2852
Jadgal, 2014		10	963	1250	2007–2011	Konarak	11		N^a^		287
Dehghan, 2013	Iran And Other part	11	545	623	2001–2011	larestan	30	256	0	2	78
Delam, 2020		12	148	190	2006–2018	Fars^b^	30				42
Ghanbarnejad, 2021		13	763	882	2011–2018	Hormozgan	N^a^				119
Hatam, 2015		14	469	803	2015	Hormozgan	N^a^				334
Purrastgu-Haghi, 2019		15	305	569	2001–2014	Haji Abad	0				264
Hanafi-Bojd, 2010		16	980	1519	2004–2008	Bandar-abbas	N^a^				539
Hanafi-Bojd, 2012		17	7071	13,490	2002–2009	Bashagard	N^a^				6419
Jaberhashemi,2018		18	772	1407	2008–2017	Bashagard	2		N^a^		635
Hosseinpoor, 2023		19	284	347	2010–2020	Jask	N^a^				63
Podat, 2006		20	4509	6905	1998–2002	Bandar Abbas	N^a^				2396
Najafi, 2006		21	458	518	2000–2004	Mazandaran	N^a^				60
Rezapour, 2022		22	591	649	2001–2020	Mazandaran	2		N^a^		58
Ghaffari, 2012		23	677	844^c^	1997–2012	Mazandaran	30		N^a^		97
Kazemi, 2016		24	208	262	1365–1388	Babol	N^a^				54
Salmanzade,2015		25	498	541	2001–2014	Khuzestan	N^a^				43
Kassiri, 2018		26	31	46	1995–2018	Gotvand	N^a^				15
Kazemi, 2018		27	34	40	2001–2016	Ramhormoz	N^a^				6
Sheikhzadeh, 2016		28	76,392	119,331	2001–2014	Endemic areas^d^	N^a^				42,939
Piroozi, 2019		29	91,914	133,886	2002–2015	Iran	N^a^		0	6	41,972
Raeisi, 2009		30	70,855	105,219	2002–2007	Iran	1087		N^a^		34,364
Norouzinejad, 2016		31	6315	7826	2011–2014	S & B and hormozgan	N^a^				1511
Nazari, 2016		32	94	168	1996–2010	Kermanshah	10		N^a^		26
Toolabi, 2016		33	74	112	2004–2014	Bam	N^a^				38
Doroudgar, 1999		34	453	498	1986–1998	Kashan	4		N^a^		45
Bafghi, 2023		35	90	95	2011–2020	Yazd	N^a^				5
Bafghi, 2013		36	179	206	2008–2012	Yazd	N^a^				27
Saberi, 2022		37	393	522	2009–2018	Kerman	N^a^				129
Mohebbi, 2018		38	161	173	2008–2017	Gheshm	N^a^				12
Najafi-sharjabad, 2022		39	663	715	2011–2018	Bushehr	N^a^				52
Sarafraz, 2016		40	115	133	2001–2013	Azerbaijan	N^a^				18
Shafiee, 2011		41	665	945	2001–2008	Khorasan Razavi	N^a^				280
Soleimanifard, 2011		42	679	726	2004–2009	Isfahan	180		N^a^		47
Fallah, 2003		43	447	506	1980–2001	Hamedan	48		N^a^		59

^a^Not Mentioned

^b^Larestan, Gerash, Evaz and Khonj in southern Fars province

^c^Gender of 15 patients were not recorded

^d^30 counties in three south-eastern provinces

**Table 3:** Methodological quality assessment through NEWCASTLE–OTTAWA scale (for cross-sectional studies)

Row	Author, year	Selection				Comparability	Outcome		score
		**Representativeness of the sample**	**Sample size**	**Non-respondent**	**Ascertainment of the exposure**	**The subjects in different outcome groups are comparable based on the study design or analysis. Confounding factors are controlled**	**Assessment of the outcome**	**Statistical test**	**0–10 star**
1	Salehi, 2010	a (*)	a (*)	c	a (**)	a (**)	a (**)	b	8
2	Salehi, 2008	a (*)	a (*)	c	a (**)	a (**)	a (**)	a (*)	9
3	Sargolzaie, 2014	a (*)	a (*)	c	b (*)	b	a (**)	b	5
4	Zayeri, 2011	a (*)	a (*)	c	a (**)	a (**)	a (**)	a (*)	9
5	Youssefi, 2011	a (*)	a (*)	c	a (**)	a (**)	a (**)	b	8
6	Mehdipour, 2013	a (*)	b	c	a (**)	a (**)	a (**)	b	7
7	Mirahmadi, 2020	a (*)	a (*)	c	a (**)	a (**)	a (**)	a (*)	9
8	Nili, 2023	a (*)	a (*)	c	a (**)	a (**)	a (**)	a (*)	9
9	Sharifi-Mood, 2012	a (*)	a (*)	c	c	a (**)	c (*)	b	5
10	Jadgal, 2014	a (*)	a (*)	c	a (**)	a (**)	a (**)	b	8
11	Dehghan, 2013	a (*)	a (*)	c	a (**)	a (**)	a (**)	a (*)	9
12	Delam, 2020	a (*)	a (*)	c	a (**)	a (**)	a (**)	a (*)	9
13	Ghanbarnejad, 2021	a (*)	a (*)	c	a (**)	a (**)	a (**)	a (*)	9
14	Hatam, 2015	b (*)	b	c	a (**)	a (**)	a (**)	a (*)	8
15	Purrastgu-Haghi, 2019	a (*)	a (*)	c	a (**)	a (**)	a (**)	b	8
16	Hanafi-Bojd, 2010	a (*)	a (*)	c	a (**)	a (**)	b (**)	b	8
17	Hanafi-Bojd, 2012	a (*)	b	c	a (**)	a (**)	b (**)	b	7
18	Jaberhashemi,2018	a (*)	a (*)	c	a (**)	a (**)	b (**)	b	8
19	Hosseinpoor, 2023	a (*)	c	c	a (**)	a (**)	a (**)	b	7
20	Podat, 2006	a (*)	c	c	a (**)	a (**)	a (**)	b	7
21	Najafi, 2006	a (*)	a (*)	c	a (**)	a (**)	a (**)	a (*)	9
22	Rezapour, 2022	a (*)	a (*)	c	a (**)	a (**)	a (**)	a (*)	9
23	Ghaffari, 2012	a (*)	a (*)	c	a (**)	a (**)	a (**)	a (*)	9
24	Kazemi, 2016	a (*)	a (*)	c	a (**)	a (**)	a (**)	a (*)	9
25	Salmanzade,2015	a (*)	a (*)	c	a (**)	a (**)	b (**)	b	8
26	Kassiri, 2018	a (*)	a (*)	c	a (**)	a (**)	a (**)	b	8
27	Kazemi, 2018	a (*)	a (*)	c	a (*)	a (**)	b (**)	b	7
28	Sheikhzadeh, 2016	a (*)	a (*)	c	a (**)	a (**)	a (**)	a (*)	9
29	Piroozi, 2019	a (*)	b	c	a (**)	a (**)	a (**)	b	7
30	Raeisi, 2009	a (*)	a (*)	c	a (**)	a (**)	a (**)	b	8
31	Norouzinejad, 2016	a (*)	a (*)	c	a (**)	a (**)	a (**)	a (*)	9
32	Nazari, 2016	a (*)	a (*)	c	a (**)	a (**)	a (**)	a (*)	9
33	Toolabi, 2016	a (*)	a (*)	c	a (**)	a (**)	a (**)	a (*)	9
34	Doroudgar, 1999	a (*)	a (*)	c	b (*)	a (**)	b (**)	b	7
35	Bafghi, 2023	a (*)	a (*)	c	a (**)	a (**)	a (**)	a (*)	9
36	Bafghi, 2013	a (*)	a (*)	c	a (**)	a (**)	b (**)	a (*)	9
37	Saberi, 2022	a (*)	a (*)	c	a (**)	b	a (**)	a (*)	7
38	Mohebbi, 2018	a (*)	a (*)	c	a (**)	a (**)	a (**)	a (*)	9
39	Najafi-sharjabad, 2022	a (*)	a (*)	c	a (**)	b	a (**)	a (*)	7
40	Sarafraz, 2016	a (*)	a (*)	c	a (**)	a (**)	a (**)	b	8
41	Shafiee, 2011	a (*)	a (*)	c	a (**)	a (**)	a (**)	b	8
42	Soleimanifard, 2011	a (*)	a (*)	c	c	a (**)	b (**)	a (*)	7
43	Fallah, 2003 [35]	a (*)	a (*)	c	a (**)	a (**)	a (**)	b	8

^*^One score

^**^Two score

### Outcome of study

#### Frequency of malaria based on Gender

**Fig. 2:**
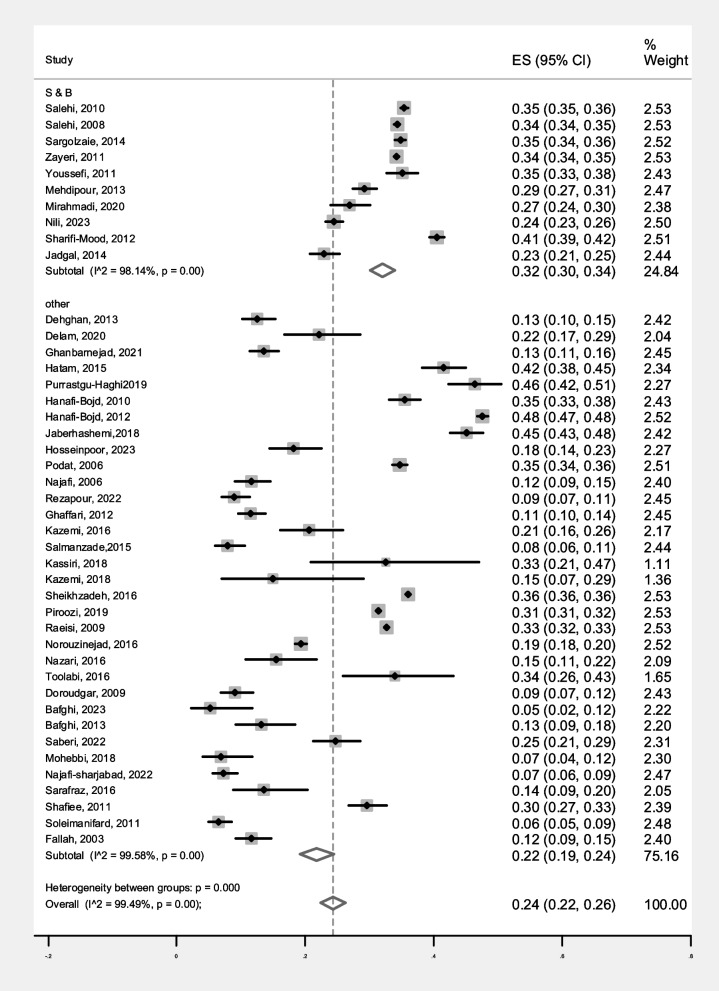
Forest plot diagram of the frequency of malaria in women of S&B and other parts of Iran from all infected people according to primary studies and overall estimate with 95% confidence interval

**Fig. 3:**
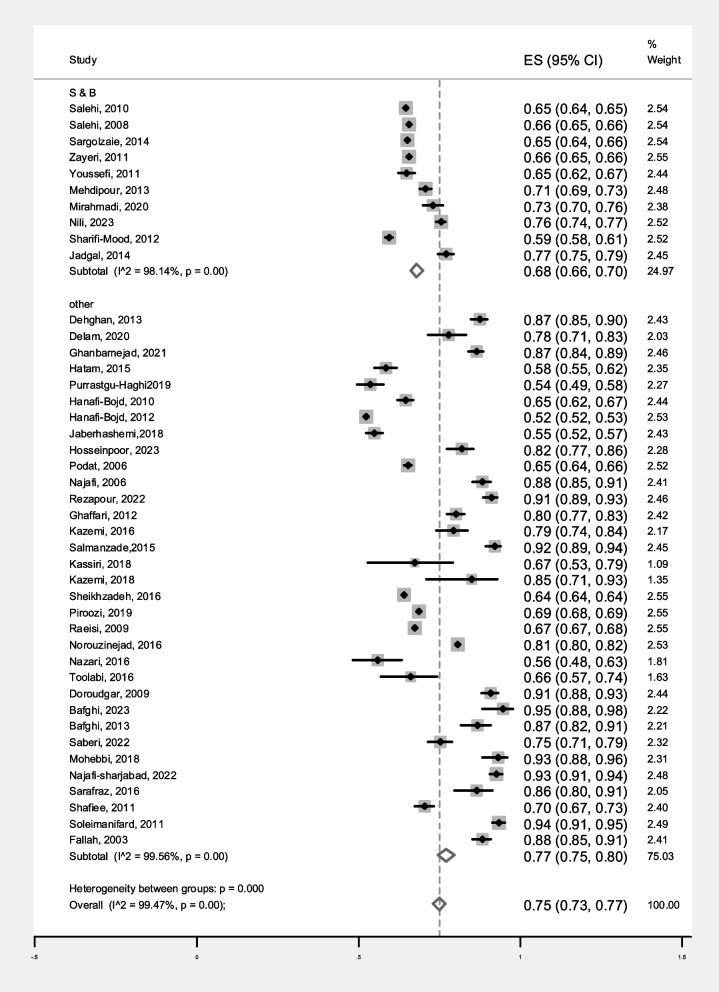
Forest plot diagram of the frequency of malaria in men of S&B and other parts of Iran from all infected people according to primary studies and overall estimate with 95% confidence interval

**Fig. 4:**
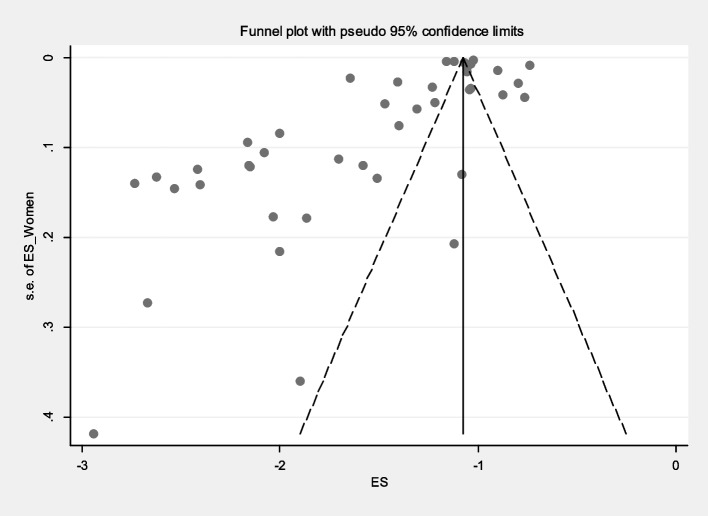
Funnel plot diagram to evaluate the publication bias in the estimation of the frequency of malaria in women among the total number of patients according to primary studies and the overall estimate with a 95% confidence interval

**Fig. 5:**
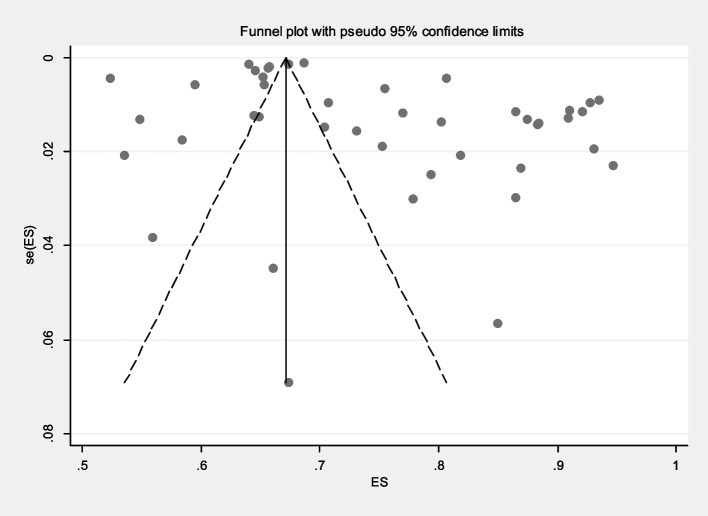
Funnel plot diagram to evaluate the publication bias in the estimation of the frequency of malaria in men among the total number of patients according to primary studies and the overall estimate with a 95% confidence interval

**Fig. 6:**
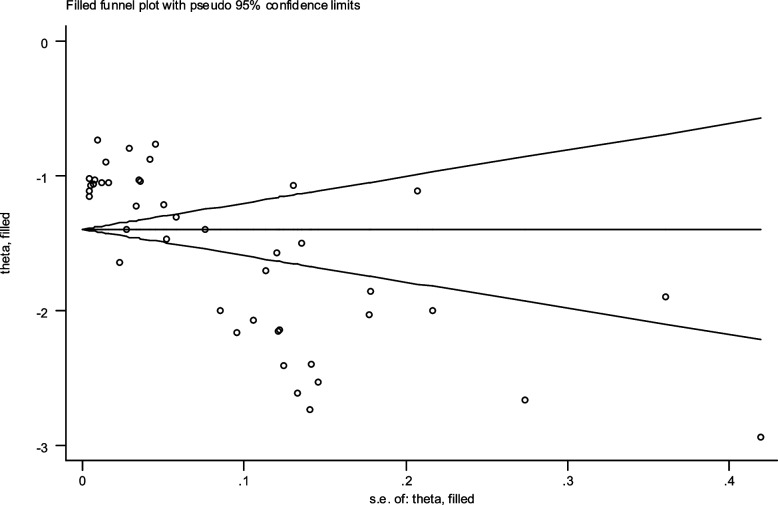
Trim and Fill diagram to estimate the number of possible studies with publication bias

The prevalence of malaria based on gender in S&B region (10 primary studies) and other regions of Iran (33 primary studies) has been reported in 43 primary studies. Heterogeneity incidence indicate significant heterogeneity between the (I-squared: 99.49%, Q: 8268.27, *P* < 0.001). By combining the results of the 43 primary studies using the random effect model, the frequency of malaria among all infected women in S&B is 32% (95% CI: 30–34%), in other part of Iran 22% (95% CI: 19–24%) and the overall estimate for Iran is 24% (95% CI: 22–26%) (Fig. 2). Likewise, the frequency of malaria among all infected men in S&B is 68% (95% CI: 66–70%), in other part of Iran 77% (95% CI: 75–80%) and the overall estimate for Iran is 75% (95% CI: 73–77%) (Fig. 3). Additionally, the funnel plot diagram (Fig. 4) and the Egger’s test results (β = -4.66, *P* = 0.013) of women and Subsequently the funnel plot diagram (Fig. 5) and the Egger’s test results (β = 7.57, *P* = 0.004) of men revealed significant publication bias in estimating the frequency of malaria based on gender. The Trim and Fill test (Fig. 6) was performed to estimate the number of possible studies with publication bias, but no new study was identified, and the results remained unchanged. It should be noted that based on the results of the sensitivity analysis, the impact of each primary studies on the prevalence of malaria in women and men was not significant.

#### Relapse and death of malaria

Among the primary studies included in the research, the death rate was investigated in two. In one of the studies examining malaria across Iran from 2002 to 2015, out 133,886 cases, 6 deaths were recorded, all involved men. Another study focused on the city of Larestan in Fars province between 2006-2018, reported 2 deaths, both in men.

**Fig. 7:**
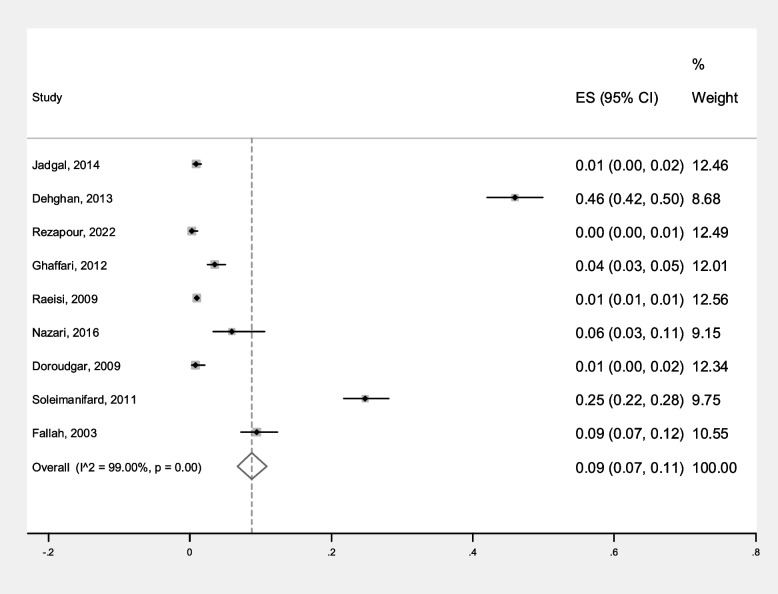
Forest plot diagram of malaria recurrence according to primary studies and overall estimate with 95% confidence interval

**Fig. 8:**
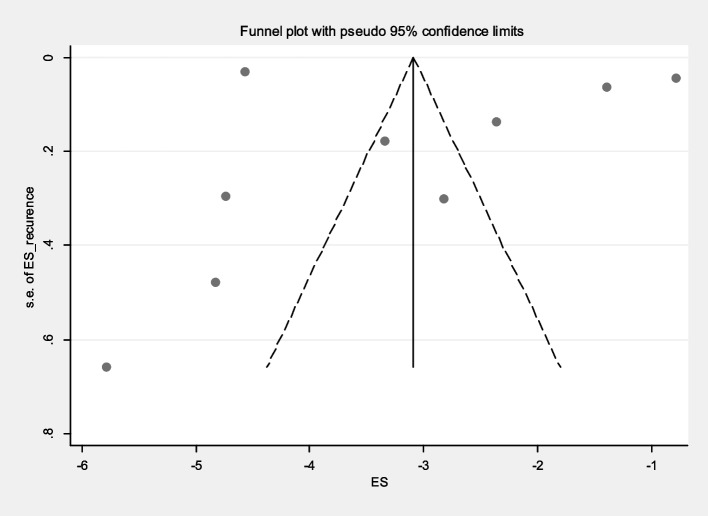
Funnel plot diagram to evaluate the publication bias in the estimation of malaria recurr

The incidence of malaria relapse was reported in 9 primary studies, varying from at least 0.3% to 46%. By combining the results of these 9 studies using the random effect model (Q: 801.60, *P*<0.001), the incidence of malaria relapse in Iran was estimated at 9% (95% CI: 7-11%) (Fig. 7). The funnel plot diagram (Fig. 8) and Egger’s test results (β=4.87, *P*=0.734) indicate that publication bias is not significant in the estimation of malaria relapse in Iran. the sensitivity analysis also shows that the impact of each of the primary studies on the incidence of malaria relapse is not significant.

#### Climate change and incidence of malaria

Regarding influencing weather conditions, out of the total 43 studies included in this study, 19 reported the time pattern of malaria incidence. the highest incidence of malaria in Iran, was reported in18 studies during spring (20 March to 20 June) and summer (21 June to 21 September). only one study reported the highest malaria prevalence in autumn (September). According to the studies, the lowest incidence of malaria, was reported in12 studies during the winter season (21 December to 19 March), and in two studies during winter and spring. Most studies showed a downward trend in malaria incidence over the study years between 1986 to 2019, indicating that the incidence was higher at the beginning and decreased over time. One study (Delam 2020) found an initial increase followed by a decrease during the study years (Table 4).

**Table 4:** The number of malarias based on the Seasonal pattern

Row	Author, year	Year of review	The most common month	The most common year	The least common month	The least common year	The most common Season	The least common season
1	Mirahmadi, 2020 [4]	2001–2016	April to September	2001	Jan And Feb	2016	Spring and summer	Winter
2	Delam, 2020	2006–2018	August	2017	Feb and march	2013	Summer	Winter
3	Purrastgu-Haghi, 2019	2001–2014	June, July	2005	-	2011–2014	Summer	Winter
4	Nazari, 2016	1996–2010	May	1996	Feb	2010	Spring	Winter
5	Najafi, 2006	2000–2004	Spring	2002	-	2004	Spring	Winter
6	Rezapour, 2022	2001–2020	May	2004	Feb	2019	Spring	Winter
7	Hanafi-Bojd, 2010	2004–2008	July	2005	Jan And Feb	2006–2007	Summer	Winter
8	Hanafi-Bojd, 2012	2002–2009	August	2007	Jan And Feb	2007–2009	Summer	Winter
9	Jaberhashemi,2018	2008–2017	June	2008	Jan And Feb	201	Spring	Winter
10	Kassiri, 2018	1995–2018	August	1995	Feb And March	2012–2018	Summer	Winter and spring
11	Shafiee, 2011	2001–2008	August	2001	Jan And March	2006	Summer	Winter and spring
12	Soleimanifard, 2011	2004–2009	May	2005	Jan	2009	Spring	Winter
13	Mohebbi, 2018	2008–2017	Sep	2011	Feb	2017	Autumn	Winter
14	Kazemi, 2016	1986–2009	June	1986	-	2005,2007,2009	Summer	-
15	Sarafraz, 2016	2001–2013	June	2003	-	2010,2013	Summer	-
16	Nili, 2023	2000–2019	May	-	Feb	-	Spring	Winter
17	Hanafi-Bojd, 2012	2000–2009	August	2007	-	-	Summer	-
18	Ghanbarnejad, 2021	2011–2018	-	2011	-	2018	Summer	-
19	Fallah, 2003	1980–2001	Juley, May	-	-	2001	Spring and summer	-

#### Socio-demographic and incidence of malaria

In terms of socio-demographic status affecting the incidence of malaria, out of the 43 studies, 19 studies reported the location of malaria patients, with 18% (29,574 cases) occurrence in urban and 82% (139,649 cases) occurrence in rural areas. out of the 19 studies, in only 5 studies the rate of infection was higher in the city than in the village. In terms of work status, 8 studies mentioned the employment status of malaria cases; 28% were employed (1298 cases), 12% were house-keeper (536 cases), and the remaining 60% were farmers, soldiers, students, children and others. In terms of the age, young and old individuals (15 to 50 years) had the highest rate of infection and individuals less than 15 and more than 50 years old, had the lowest rate of infection (Table 5).

**Table 5:** The number of malarias according to socio-demographic variables

Author, year	Row	Residence place		Total case of malaria	Age		Job status		
		**Rural**	**Urban**		**lowest rate**	**highest rate**	**Other**^**b**^	**House-keeper**	**Worker**
Mirahmadi, 2020 [4]	1	426	376	802	-	> 60	463	127	212
Delam, 2020	2	45	145	190	45–49	20–24	48	18	124
Ghanbarnejad, 2021	3	429	453	882	> 65	16–29	363	40	479
Bafghi, 2023	4	N^*^		95	5-19y	30-39y	26		69
Jaberhashemi,2018	5	1397	10	1407	26–35	6-15y	1158	179	70
Kassiri, 2018	6	N^a^		46	> 60	20–29	19	8	19
Mohebbi, 2018	7	31	142	173	10-Jun	21–30	22	-	151
Shafiee, 2011	8	N^a^		945	< 5y	> 15	607	164	174
Hanafi-Bojd, 2010	9	977	542	1519	< 5y	> 14y	N^a^		
Zayeri, 2011 [36]	10	55,799	9127	64,920	< 5y	> 15	N^a^		
Fallah, 1382	11	298	208	506	< 9y	20–29	N^a^		
Hosseinpoor, 2023	12	300	47	347	< 5y	> 14y	N^a^		
Kazemi, 1395	13	202	60	262	N^*^	> 15	N^a^		
Podat, 1385	14	4285	2620	6905	< 5	> 15	N^a^		
Salehi, 2010	15	22,670	5502	28,172	< 4y	15–44	N^a^		
Nili, 2023	16	1018	3056	4074	< 5	> 15	N^a^		
Salehi, 2008	17	38,325	3837	42,162	< 5	> 15	N^a^		
Youssefi, 2011	18	1335	129	1464	> 14	< 5	N^a^		
Dehghan, 2013	19	N^*^		623	< 1y	20-29y	N^a^		
Doroudgar, 1378	20	N^*^		498	> 50	20–29	N^a^		
Purrastgu-Haghi, 2019	21	N^*^		569	< 5	> 15	N^a^		
Rezapour, 2022	22	N^*^		649	> 70	20–29	N^a^		
Hatam, 2015	23	N^*^		803	> 51	21–30	N^a^		
Salmanzade,2015	24	N^*^		541	< 5y	> 15	N^a^		
Sheikhzadeh, 2016	25	N^*^		119,331	N^*^	> 15	N^a^		
Ghaffari, 2012	26	N^*^		844	> 61y	21-30y	N^a^		
Hanafi-Bojd, 2012	27	N^*^		13,490	< 5y	> 14y	N^a^		
Najafi-sharjabad, 2022	28	N^*^		715	> 50	20–29	N^a^		
Sarafraz, 2016	29	N^*^		133	< 20	31–40	N^a^		
Raeisi, 1388	30	N^*^		105,219	< 5y	> 15	N^a^		
Bafghi, 2013	31	N^*^		206	< 10y	31–40	N^a^		
Jadgal, 1393	32	963	287	1250	N^a^		N^a^		
Sargolzaie, 2014	33	10,882	2738	13,620	N^a^		N^a^		
Kazemi, 2018	34	34	6	40	N^a^		N^a^		
Saberi, 2022	35	233	289	522	N^a^		N^a^		
**Total**		**139,649**	**29,574**	**413,924**	**-**	**-**	**2706**	**536**	**1298**

^a^Not Mentioned

^b^other: child, student, farmer, Rancher, etc

## Discussion

This systematic review and meta-analysis investigated the gender characteristics, social determinants, seasonal pattern of malaria incidence, relapse, and mortality in the S&B province and other regions of Iran. A total of 43 studies were included that 10 of them was in S&B and other was from Iran and another province of Iran.

The combined results of the 43 primary studies using the random effect model showed that the frequency of malaria among infected women is 32% in S&B, 22% in other parts of Iran and 24% in the whole of Iran. This is 68%, 77% and 75% in men respectively. Our findings are in line with those of Umaru et al. (2015) who also reported a lower incidence of malaria in women compared to men [27]. Another study indicated that, women seeking treatment for fever and malaria were almost twice as many as men, suggesting a higher number of women with malaria [28], however, this might be due to women utilizing health services for treatment more than men, not a higher incidence of malaria in women. Although, the mentioned study showed that the probability of a positive malaria test was lower in women than men, that is consistent with our findings and so other studies [29–35]. Mirahmadi et al. (2020) [4], zayeri et al (2011) [36] and abdalla et al. (2007) [37] demonstrate that the incidence of malaria are lower in women than men, that are align with our study. The difference in infection rates between the gender may be attributed to biological differences or social behavioral differences for example social activities that increase exposure to vector mosquitoes. In contrast, a study conducted by Okwa et al. (2010) in Nigeria found that women were more likely to have malaria than men [38], This could be due to the type of Islamic clothing worn by women, who are less likely to be bitten by insects. Therefore, since the risk of infection is probably same in both male and female, prevention and treatment strategies need to be implemented in both sexes.

Our study showed that, among the primary studies included in the research, 6 deaths were recorded in one study, and 2 death in another study, all of whom were men. Malaria epidemics cause significant morbidity and often mortality where they occur [39, 40]. One study in Sudan show that males had the highest incidence and mortality than women [37]. Death from malaria can be due to people seeking health care late or not receiving proper treatment [41], this issue is seen more in men than women and could possibly explain the higher incidence of death in men. Although malaria morbidity and mortality are decreasing, it remains an important health issue [33], because it can significantly reduce the disease burden.

The results of this systematic review and meta-analysis also indicate that the incidence rate of malaria relapse varied from at least 0.3% to 46%. Based on the random effect model, the incidence of malaria relapse in Iran was estimated to be 9%. Kwak et al. (2013) showed the relapse rate of vivax malaria was 3.2% [42]. Moon et al. (2009) reported that of the 3881 reported malaria cases (2375 soldiers and 1506 veterans), 62 cases (1.6%) experienced a second attack and 2 cases (0.05%) experienced a third attack [43]. In a study of Saifi et al. (2010) relapse rate was also low (i.e. 4.5%) [44]. The low recurrence rate indicates an appropriate surveillance, control, and prevention system for malaria. Although according to WHO’s recommendation to prevent re-emergence in malaria-free areas and elimination in areas where malaria is limited, it is necessary to take appropriate measures to prevent the relapse of this disease.

In terms of influencing weather conditions, our study is in line with other study showing that malaria incidence fluctuates during different months and seasons [45–47]. Winter is too cold for malaria to be transmissible, and temperature and humidity are usually not favorable until April [48], consistent with our findings. Zhang et al. (2010) showed that 1°C rise in maximum temperature may be related to a 7.7% to 12.7% increase and a 1°C rise in minimum temperature may result in approximately 11.8% to 15.8% increase in the number of malaria cases [49]. Other studies have also confirmed the connection between heat and the incidence of malaria [32, 33, 50–52]. Temperature affects the sporogony cycle of the plasmodium and the longevity of the vector [6, 53]. In addition, a 1°C increase in air temperature was predicted to reduce the larval duration of anopheles gambiae from 47 to 37 days. It can be concluded that temperature affects every stage of the mosquito’s life [6] leading to an increase in malaria rates, one reasons for the higher incidence of malaria in S&B can be attributed to favorable weather conditions for mosquitoes. On the other hand, in many provinces of Iran, we are observed an increase in the number of villages covered by electricity. Therefore, benefiting from electricity and using cooling devices has meant that even in the hot seasons of the year and despite the increase in air temperature, people can sleep indoors at night, and so avoid mosquito bites and break the chain of malaria transmission [54].

In addition, most studies showed a downward trend in the incidence of malaria over the study years. In line with this, another study reported a decline in malaria cases in Iran from 1847 to 81 between 2010 and 2017 [48]. Other studies also showed overall decline in malaria from 2000 to 2012 [55, 56]. This decrease can be attributed to malaria care program, the efficiency of health systems and a decrease in the number of immigrants. However, one study within our research showed an increase in malaria infection, possibly due to the re-emergence of malaria. Factors for this increase include the rising number of malaria discovery laboratory tests, increased rainfall in recent years providing suitable conditions for anopheles growth and proliferation, favorable temperature and humidity, and an increasing number of refugees and displaced persons from neighboring countries. Tesfay et al. (2018) showed that the overall trends of malaria case increased over the past 6 years (2011–2016) in Raya Azebo district, Northern Ethiopia [57]. This may be due to more detection and more reporting of positive cases. However, strong effort is needed to improve malaria prevention and control method in area like Ethiopia. These findings are confirmed by a study of Masaninga et al. (2013) which showed that, despite a decline in malaria disease burden over the past decade in Zambia, a reversal in impact was notable in the years 2009–2010, indicating that control gains are fragile and must be sustained to eliminate malaria [58]. Therefore, it seems that in most countries there is always a need for malaria eradication programs, dedicated health personnel to combat malaria, and financial resources to combat malaria.

In this systematic review, the socio-demographic status affecting the incidence of malaria was also assessed. although current evidence-based documents regarding the effect socio-demographic status on the incidence of malaria are limited, the findings showed that malaria is more common in rural areas than in urban areas. of 19 studies, only 5 reported a higher infection rate in cities than in villages. Other studies also demonstrated the relationship between the socio-economic class, place of residence and malaria incidence [27, 59–63]. This could be due to people with higher socio-economic status having more knowledge to prevent malaria. The higher incidence in rural areas may be due to less access to health facilities and protective equipment, such as nets. Additionally, living in the rural areas provides suitable conditions for mosquito breeding due to increased agricultural activities. Our study also revealed that young to middle-aged people are more likely to be exposed to malaria. In line with this, a study showed that malaria is more common in older children (15–17 years old) [64]. Other studies confirmed our findings regarding the age of patients with malaria [34, 35, 65, 66]. This can be explained by the fact that this age group is more likely to engage in outdoor activities or travel to other areas. To our knowledge, this is the first systematic review and meta-analysis in this field conducted in Iran.

### Strengths and limitations

#### Strengths

This study has used a systematic approach to search in multiple databases, which increases the strength of the findings. In addition, the Newcastle-Ottawa tool was used to check the quality of the studies, which gives credibility to the findings. furthermore, meta-analysis by combining data provides a suitable conclusion of evidence.

#### Limitations

The heterogeneity observed among the included studies may introduce variability in the results. The absence of some information in some studies made it impossible to conduct a meta-analysis. Although, A significant percentage of malaria cases are imported, that have great epidemiological importance, but in this systematic review and meta-analysis, it was not possible to separate imported and local cases. The reason for this limitation was that malaria cases were not reported separately from local and imported cases in most of the initial studies.

## Conclusion

This systematic review and meta-analysis revealed that the incidence of malaria in women was lower than in men. The highest incidence of malaria in Iran was between April to September while the lowest incidence was between January to March, showing a downward trend over the study years. Malaria is more common in rural areas, and young to middle-aged people are more likely to be exposed. According to the findings, there is still a need for health care, and planning for appropriate malaria control interventions especially for men, rural areas, hot weather, and young to middle-aged age groups is necessary for the elimination of malaria, particularly in S&B are necessary.

## Supplementary Information

Supplementary Material 1.

## Data Availability

Data is provided within the manuscript or supplementary information files.
