# Supplementary material for: Gender characteristics, social determinants, and seasonal patterns of malaria incidence, relapse, and mortality in Sistan and Baluchistan province and other province of Iran: A systematic review and meta-analysis
Source: BMC Infect Dis. 2025 Feb 1;25:154. doi: 10.1186/s12879-025-10542-0 (PMC11787763; doi:10.1186/s12879-025-10542-0)
Supplement: Supplementary file 1 — Supplementary Material 1. [file 12879_2025_10542_MOESM1_ESM.docx]

**Supplementary file**

**Search strategy in databases**

| Database | Search strategy | Search filter |
| --- | --- | --- |
| PubMed | (((((((Marsh Fever[Title/Abstract]) OR (Fever, Marsh[Title/Abstract])) OR (Remittent Fever[Title/Abstract])) OR (Fever, Remittent[Title/Abstract])) OR (Infections, Plasmodium[Title/Abstract])) OR (Infection, Plasmodium[Title/Abstract])) OR (Plasmodium Infection[Title/Abstract])) OR (Plasmodium Infections[Title/Abstract])) OR (Malaria[Title/Abstract]) AND (Islamic Republic of Iran[Title/Abstract]) OR (Iran[Title/Abstract]) AND (Gender Identity[Title/Abstract]) OR (Sex[Title/Abstract]) AND (((((((Change, Climate[Title/Abstract]) OR (Changes, Climate[Title/Abstract])) OR (Climate Changes[Title/Abstract])) OR (Climate Change[Title/Abstract])) OR (Temperature[Title/Abstract])) OR (Humidity[Title/Abstract])) OR (Atmospheric pressure[Title/Abstract])) OR (Wind[Title/Abstract]) | - Species: Human - Article Language: English, Persian - Article type: All types of studies except Case reports, case series and RCTs |
| Scopus | (TITLE-ABS-KEY (marsh AND fever) OR TITLE-ABS-KEY (fever, AND marsh) OR TITLE-ABS-KEY (remittent AND fever) OR TITLE-ABS-KEY (fever, AND remittent) OR TITLE-ABS-KEY (infections, AND plasmodium) OR TITLE-ABS-KEY (infection, AND plasmodium) OR TITLE-ABS-KEY (plasmodium AND infection) OR TITLE-ABS-KEY (plasmodium AND infections) OR TITLE-ABS-KEY (malaria)) AND (TITLE-ABS-KEY (Islamic AND republic AND of AND Iran) OR TITLE-ABS-KEY (Iran)) | - Subject areas: Medicine - Document types: Article - Source type: Journal |
| WOS | ((((((((TS= (Marsh Fever)) OR TS= (Fever, Marsh)) OR TS= (Remittent Fever)) OR TS= (Fever, Remittent)) OR TS= (Infections, Plasmodium)) OR TS= (Infection, Plasmodium)) OR TS= (Plasmodium Infection)) OR TS= (Plasmodium Infections)) OR TS=(Malaria) AND (TS= (Islamic Republic of Iran)) OR TS=(Iran) AND (TS=Gender Identity) OR (TS=Sex)  (((((((TS=(Change, Climate)) OR TS=(Changes, Climate)) OR TS=(Climate Changes)) OR TS=(Climate Change)) OR TS=(Temperature)) OR TS=(Humidity)) OR TS=(Atmospheric pressure)) OR TS=( Wind) | - Document types: Article |
| ScienceDirect | Marsh Fever OR Fever, Marsh OR Remittent Fever OR Fever, Remittent OR Infections, Plasmodium OR Infection, Plasmodium OR Plasmodium Infection OR Plasmodium Infections OR Malaria AND Islamic Republic of Iran OR Iran AND Gender Identity OR Sex AND Change, Climate OR Changes, Climate OR Climate Changes OR Climate Change OR Temperature OR Humidity OR Atmospheric pressure OR Wind | - Article type: Research article - Access type: Open access & Open archive |
| Google Scholar | “Marsh Fever” OR Malaria AND Islamic Republic of Iran OR Iran AND “Gender Identity” OR Sex AND “Climate Change” OR Temperature OR Humidity OR Atmospheric pressure OR Wind | - Year of publication: 1990-2024 - Sort by relevance - Keywords anywhere in the article |
| SID | Malaria AND Iran AND Gender AND Climate Change | No search filters were applied |
| Magi ran | Malaria AND Iran AND Gender AND Climate Change | No search filters were applied |

**Tables based on cumulative sort**

Figure 2- Forest plot diagram of the frequency of malaria in women of S&B and other parts of Iran from all infected people according to primary studies and overall estimate with 95% confidence interval.

Figure 4- Forest plot diagram of the frequency of malaria in men of S&B and other parts of Iran from all infected people according to primary studies and overall estimate with 95% confidence interval.

Figure 7- Forest plot diagram of malaria recurrence according to primary studies and overall estimate with 95% confidence interval
